# Pediatric T-Cell/Histiocyte-Rich Large B-Cell Lymphoma (THRLBC) in an 8-Year-Old Male Child: A Case Report

**DOI:** 10.1155/crom/8869045

**Published:** 2025-02-22

**Authors:** Gashaw Arega, Haileyesus Adam, Alemayehu Girma, Galgaloo Diida, Eden Beresa, Leul Adane, Michael A. Negussie, Fadil Nuredin Abrar, Mesfin Asefa

**Affiliations:** ^1^Division of Hematology and Oncology, Department of Pediatrics and Child Health, College of Health Sciences, Addis Ababa University, Addis Ababa, Ethiopia; ^2^Department of Radiology, College of Health Sciences, Addis Ababa University, Addis Ababa, Ethiopia; ^3^School of Medicine, College of Health Sciences, Addis Ababa University, Addis Ababa, Ethiopia; ^4^Department of Pathology, College of Health Sciences, Addis Ababa University, Addis Ababa, Ethiopia; ^5^Department of Pathology, St. Paul's Hospital Millennium Medical College, Addis Ababa, Ethiopia; ^6^ONCO Pathology Diagnostic Center, Addis Ababa, Ethiopia

**Keywords:** chemotherapy, diffuse large B-cell lymphoma, immunohistochemistry, pediatric oncology, T-cell/histiocyte-rich large B-cell lymphoma

## Abstract

T-cell/histiocyte-rich large B-cell lymphoma (THRLBCL) is a rare and aggressive subtype of diffuse large B-cell lymphoma (DLBCL) that is uncommon in children. Here, we present the case of an 8-year-old male with a 3-month history of low-grade intermittent fever, significant weight loss, loss of appetite, and progressive abdominal swelling. Examination revealed splenomegaly and a palpable midabdominal mass, with laboratory findings showing bicytopenia. Imaging demonstrated hepatosplenomegaly, diffuse hypodense liver and spleen lesions, and mesenteric and retroperitoneal lymphadenopathy. A core-needle biopsy of the mesenteric mass confirmed the diagnosis, with histopathology revealing scattered large mononuclear and binucleate cells in a background of small lymphocytes and histiocytes. Immunohistochemistry showed positivity for CD45, CD20, and EMA and negativity for CD30, CD15, and Bcl-2, excluding alternative diagnoses such as nodular lymphocyte-predominant Hodgkin lymphoma (NLPHL) and classical Hodgkin lymphoma (cHL). The patient was initially stabilized with a prephase regimen of cyclophosphamide, vincristine, and prednisone (COP), followed by induction and consolidation with R-COPADM (rituximab, cyclophosphamide, vincristine, prednisone, and methotrexate). Posttreatment imaging revealed significant resolution of lymphadenopathy and hepatosplenomegaly, with no residual or recurrent disease. At follow-up, the patient remains in clinical remission with no signs of progression. This case highlights the importance of early recognition, detailed histopathological evaluation, and the role of immunohistochemistry in accurately diagnosing THRLBCL in children, ensuring timely initiation of effective therapy and improving outcomes in this rare pediatric malignancy.

## 1. Introduction

T-cell/histiocyte-rich large B-cell lymphoma (THRLBCL) is a subtype of diffuse large B-cell lymphoma (DLBCL) characterized by scattered neoplastic large B-cells with predominantly small reactive T-cells and histiocytes in the background, along with an absence of follicular dendritic mesh works [1]. THRLBCL is most commonly observed in older individuals and is rarely diagnosed in the pediatric population. Misdiagnosis as classical Hodgkin lymphoma (cHL) or nodular lymphocyte-predominant Hodgkin lymphoma (NLPHL) poses a significant diagnostic challenge due to overlapping histological features [2–4].

This report describes a rare pediatric case of THRLBCL, focusing on the clinical presentation, diagnostic process, and therapeutic outcomes, providing valuable insights into the recognition and management of this uncommon malignancy in children.

## 2. Case Presentation

A previously healthy 8-year-old boy developed a 1-week history of low-grade intermittent fever, loss of appetite, and weight loss 3 months prior to presentation at our facility. Initially, he was treated at a local health center with paracetamol and antibiotics for a presumed infection. However, his symptoms persisted, and over the following weeks, he developed progressive abdominal swelling. With no history of chronic cough, night sweats, or known exposure to tuberculosis, his condition continued to worsen, prompting referral to our facility for further evaluation and management.

At presentation, the patient appeared active, with normal vital signs. Cardiovascular and chest examinations were normal. Abdominal examination revealed splenomegaly, with the spleen palpable 7 cm below the costal margin, firm, and nontender. A 3 × 2 cm firm, distinct midabdominal mass was noted. There was no peripheral lymphadenopathy, jaundice, or rash. Musculoskeletal and neurological examinations were unremarkable.

**Figure 1:**
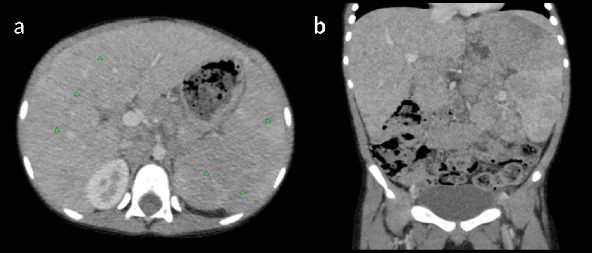
Prechemotherapy postcontrast (portal venous phase) (a) axial and reconstructed (b) coronal CT images demonstrate hepatosplenomegaly, with the liver and spleen measurements of 17.4 and 17.3 cm, respectively. Both organs show heterogeneous parenchymal enhancement. Arrowheads indicate diffuse hypoechoic parenchymal lesions within the liver and spleen.

**Figure 2:**
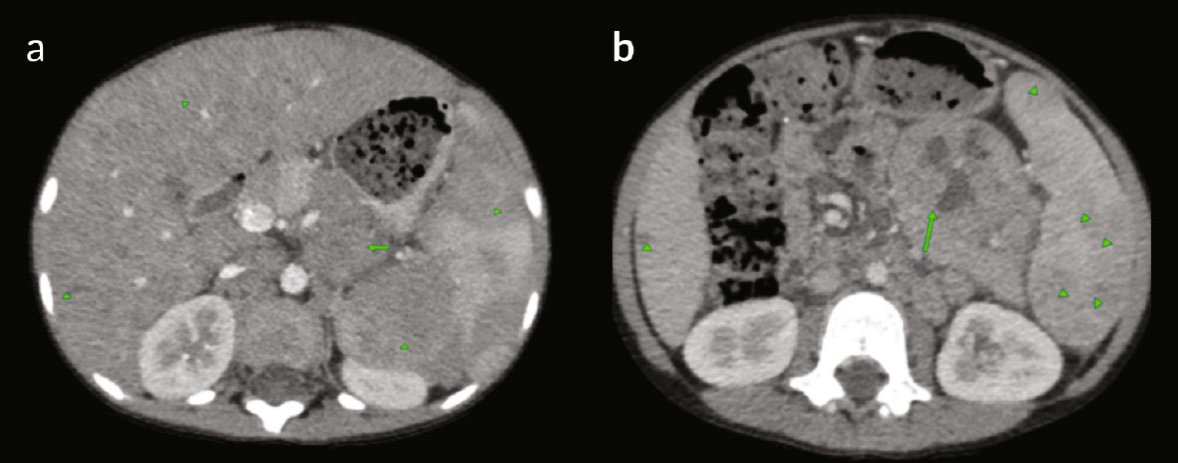
Prechemotherapy postcontrast axial CT images. (a) Diffuse hepatosplenic parenchymal lesions (arrowhead) and a 4 × 5 cm homogeneously hypodense preaortic lesion. (b) A 5 × 7.5 cm mesenteric heterogeneously enhancing mass with central necrosis.

**Figure 3:**
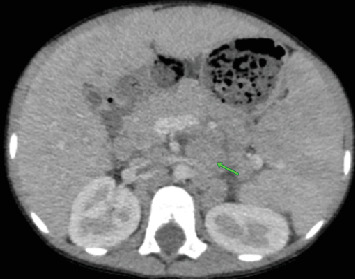
Prechemotherapy axial CT image showing a 1.2 × 2.8 cm homogeneously enhancing retroperitoneal lymph node (arrow).

Laboratory investigations revealed bicytopenia, with a white blood cell (WBC) count of 0.63 × 10^3^/*μ*L and a platelet count of 83 × 10^3^/*μ*L. Hemoglobin was within normal limits at 13.4 g/dL. A neck, chest, and abdominal CT scan was performed, revealing normal findings in the neck and chest. The abdominal CT scan showed marked hepatosplenomegaly, with heterogeneous parenchymal enhancement of both the liver and spleen. The liver measured up to 17.4 cm, and the spleen was enlarged to 17.3 cm (Figure 1). Hypodense parenchymal lesions were noted diffusely in both organs. In addition, precontrast CT images demonstrated a homogeneously hypodense preaortic lesion measuring approximately 4 × 5 cm and a heterogeneously enhancing mesenteric mass measuring 5 × 7.5 cm with central necrosis (Figure 2). Further evaluation identified a 1.2 × 2.8 cm retroperitoneal lymph node with homogeneous enhancement (Figure 3). No free intraperitoneal fluid was observed. PET-CT examination was not done. There was no evidence of gastrointestinal mucosal involvement, and endoscopy was not performed as well.

**Figure 4:**
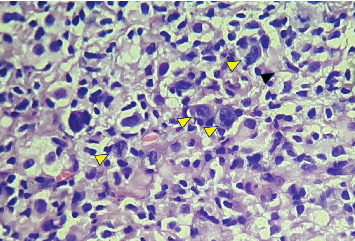
H&E stain at 40x magnification showing scattered large neoplastic cells (yellow arrowheads) within a small lymphocyte and histiocyte-rich background.

Histopathological examination was conducted on a core-needle biopsy sample taken from the mesenteric mass. It revealed a small core tissue fragment containing scattered large mononuclear and occasional binucleate cells with vesicular nuclei and prominent nucleoli, along with a few Hodgkin-like cells (Figure 4). These cells were set within a background rich in small lymphocytes and histiocytes.

**Figure 5:**
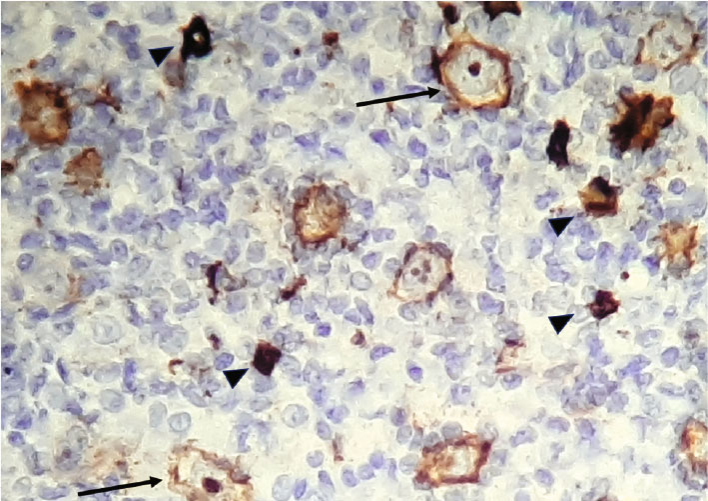
CD20 immunohistochemistry at 40x magnification highlighting the large neoplastic B-cells (thin arrow) and a few remnant reactive B-cells (arrowhead).

**Figure 6:**
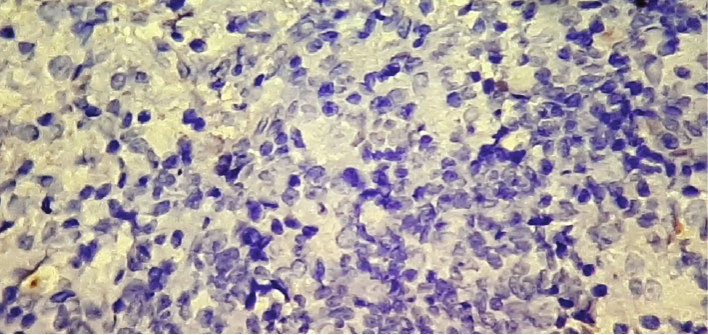
CD30 immunohistochemistry at 40x magnification showing negative staining.

Immunohistochemistry was performed with a limited panel of antibodies, as requested by the pediatric hemato-oncologist. CD45 highlighted the entire cell population, including the large cells, while CD20 selectively stained the large cells, leaving the small lymphocytes, and histiocytes in the background unstained (Figure 5). BCL2 was positive in the background small lymphocytes but negative in the large cells. CD30, CD15, EMA, TdT, and ALK1 were all negative in the large neoplastic cells (Figure 6).

**Figure 7:**
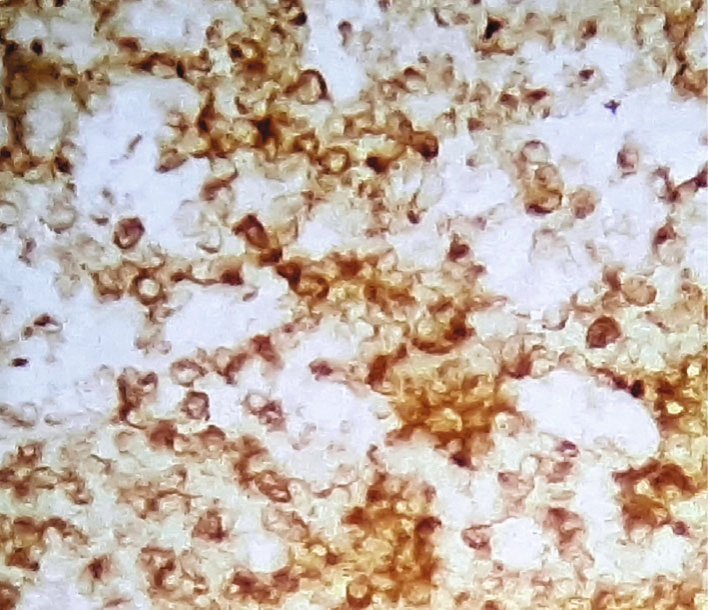
BCL2 immunohistochemistry at 40x magnification showing negative staining in the large neoplastic cells, with positive staining primarily in the background small lymphocytes.

Due to tissue depletion, additional immunohistochemistry was not possible. However, the combination of CD45 and CD20 positivity, along with the negativity for BCL2, EMA, CD30, and CD15 in the large cells, strongly supported a diagnosis of THRLBCL over alternatives such as NLPHL and cHL (Figure 7).

The patient was started on a prephase regimen of cyclophosphamide, vincristine (Oncovin), and prednisone (COP) for stabilization. Subsequently, he was transitioned to R-COPADM (rituximab, cyclophosphamide, vincristine, prednisone, and methotrexate) for induction and consolidation therapy, following standard pediatric hemato-oncology protocols.

**Figure 8:**
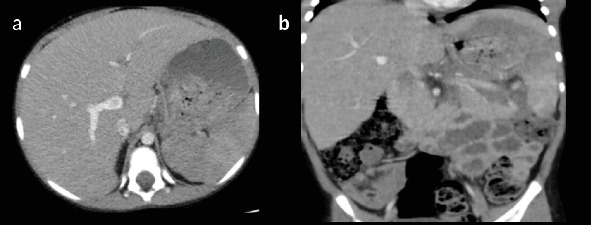
Postchemotherapy axial CT images. (a) Homogeneous liver parenchymal enhancement. (b) Normalization of splenic size to 12.7 cm.

Follow-up imaging after induction therapy demonstrated significant clinical and radiologic improvement. The liver and spleen returned to normal size with homogeneous parenchymal texture. Intra-abdominal lymphadenopathy was resolved and the pleural-based nodule regressed. No residual or recurrent masses were identified (Figure 8).

The patient continues to receive close follow-up at the pediatric hemato-oncology clinic. He is currently in clinical remission with no signs of disease progression and remains in good health.

## 3. Discussion

Malignant lymphomas, including Hodgkin lymphoma (HL) and non-Hodgkin lymphoma (NHL), are the third most common malignancy in children. DLBCL, a type of NHL, includes several subtypes, one of which is THRLBCL. THRLBCL is characterized by scattered neoplastic B-cells, which make up less than 10% of the total cells, along with numerous reactive small T-cells and histiocytes [5, 6]. Unlike other types of DLBCL, patients with THRLBCL often present with more advanced-stage disease, B symptoms, and extranodal involvement. Although THRLBCL is more common in younger adults, it is uncommon in children [2, 5, 7]. Diagnosing THRLBCL can be challenging as it may be confused with NLPHL, HL (especially the mixed cellularity type), peripheral T-cell lymphomas, and reactive processes. THRLBCL mostly affects younger adult males, with rare cases observed in children.

On the other hand, NLPHL cases are usually diagnosed at a lower stage, with most pediatric patients presenting with isolated cervical or inguinal lymphadenopathy [2, 6, 8]. Accurate diagnosis of THRLBCL relies on careful immunohistochemical analysis of tumor cells and the inflammatory microenvironment. Immunophenotyping, including a panel of immunostains for CD45, CD20, CD3, CD30, and CD15, can help distinguish THRLBCL from HL in most cases. Additional markers like BOB1 and OCT2 are used to confirm the diagnosis in difficult cases [8, 9]. THRLBCL typically shows positivity for Bcl-6 and Bcl-2 and negativity for cHL. The absence of large cells positive for CD20 and CD45 and negative for CD30 and Bcl-2 in our case makes a diagnosis of cHL less likely.

TdT and ALK were also negative. The background cellular infiltrate is important in differentiating THRLBCL from cHL, as most HL cases show a significant number of small lymphocytes and other inflammatory cells, which is uncommon in THRLBCL. EMA positivity in the large atypical cells further rules out cHL. Distinguishing THRLBCL from NLPHL can be challenging due to histological similarities, but differences in architectural background and pattern of involvement aid in differentiation [1, 10, 11]. THRLBCL is often under-recognized in children due to a higher incidence of HL in this age group and the histological resemblance between the two. Bone marrow involvement by THRLBCL can be missed on aspirate histology alone. Therefore, a high degree of suspicion and careful immunohistochemical analysis of any atypical lymphoid infiltrate involving the bone marrow is crucial for an accurate diagnosis [3, 12]. THRLBCL is a more aggressive disease, with approximately two-thirds of cases being Stage III or IV at diagnosis.

Hepatic involvement in DLBCL varies from solitary masses to diffuse infiltration, requiring imaging and histopathology for diagnosis. Han et al. [13] reported a case of primary hepatic large B-cell lymphoma (PHLBCL) presenting as a well-defined solitary mass, whereas our case of THRLBCL showed diffuse hepatosplenic involvement with multiple hypodense lesions and extensive lymphadenopathy. Both cases exhibited CD20 positivity. While their patient achieved remission with R-CHOP, ours required an intensified R-COPADM regimen due to extensive disease, highlighting the heterogeneity of hepatic lymphoma and the need for tailored management.

Treatment typically involves anthracycline-containing chemotherapy appropriate for DLBCL at a similar clinical stage. The 3-year survival rate is estimated to be 50%–64%. However, children and adolescents with THRLBCL tend to have a better prognosis and respond better to therapy compared to adults [12, 14–17].

## 4. Conclusion

Pediatric THRLBCL is rare and it poses diagnostic challenges due to overlapping features with HL and other lymphomas. Accurate diagnosis requires detailed histopathological evaluation and immunohistochemical analysis. Timely diagnosis and treatment with targeted chemotherapy can lead to remission and favorable outcomes in pediatric patients.

## Data Availability

The data supporting the findings of this case report are available from the corresponding author upon reasonable request.
